# Factors Influencing Private Hospitals’ Participation in the Innovation of Biomedical Engineering Industry: A Perspective of Evolutionary Game Theory

**DOI:** 10.3390/ijerph17207442

**Published:** 2020-10-13

**Authors:** Weiwei Liu, Jianing Yang, Kexin Bi

**Affiliations:** 1 School of Economics and Management, Harbin Engineering University, Harbin 150001, China; yjnayy0811@hrbeu.edu.cn (J.Y.); bikexin@hrbeu.edu.cn (K.B.); 2 Management School, Queen’s University Belfast, Belfast BT9 5EE, UK; 3 School of Management, Harbin University of Science and Technology, Harbin 150080, China

**Keywords:** collaborative innovation, biomedical engineering, evolutionary game theory, private hospitals

## Abstract

The innovation of the biomedical engineering (BME) industry is inseparable from its cooperation with medical institutions. China has considerable medical institutions. Although private hospitals account for more than half of Chinese medical institutions, they rarely participate in biomedical engineering industry innovation. This paper analyzed the collaborative relationship among biomedical engineering enterprises, universities, research institutes, public hospitals and private hospitals through evolutionary game theory and discussed the influence of different factors on the collaborative innovation among them. A tripartite evolutionary game model is established which regards private hospitals as a stakeholder. The results show that (1) the good credit of private hospitals has a positive effect on their participation in collaborative innovation; (2) it is helpful for BME collaborative innovation to enhance the collaborative innovation ability of partners; (3) the novelty of innovation projects has an impact on BME collaborative innovation. The specific impacts depend on the revenue, cost and risk allocation ratio of innovation partners; (4) the higher the practicability of innovation projects, the more conducive to collaborative innovation.

## 1. Introduction

In the past decade, the global biomedical engineering (BME) industry has accelerated its innovation [1]. The scale of China’s BME industry is also expanding at an alarming speed. Behind the rapid development of the industry, there is a problem worthy of consideration; that is, the gap between the level of China’s BME industrial technology and that of developed countries is obvious. This gap can be clearly shown in the import and export data of medical devices. According to the statistics of China Customs on China’s medical device import and export trade data in 2019, it is not difficult to find that the products exported by domestic BME enterprises mainly focus on low-end products with low added value, as shown in Table 1. In addition, Table 2 shows the import and export data of some high-end medical devices in China in 2019 (the data in Table 1 and Table 2 are both from the open data of the General Administration of Customs, China). Obviously, these high-tech medical device markets in China are almost monopolized by foreign-funded enterprises. Realizing the efficient innovation of the BME industry in order to narrow the technological gap with developed countries has become an urgent problem for the Chinese government at this stage.

Chen Baolin (Chen Baolin. The regional product manager of Boston Scientific, the executive committee member of China Cardiovascular Innovation (CCI) Club and the co-founder of Suzhou Endu Medical Technology Co., Ltd., Suzhou, China) pointed out that the innovation ability in the field of medical devices in China is mainly based on tracking and copying, and the collaborative innovation mechanism of medical research and enterprises needs to be improved. Collaborative innovation is an innovation mode, with knowledge value-added as the core, including enterprises, governments, universities, scientific research institutions, intermediary institutions and users as the main body [2]. This innovation mode can integrate innovation resources, promote complementary advantages between innovation subjects and accelerate the industrialization and application of innovative technology. This innovation theory has been suggested by the government and scholars to apply to BME industry innovation.

Different from other industries, hospitals, which are also one of the stakeholders, should also be considered in the collaborative innovation system due to the particularity of the BME industry [3]. In the process of BME innovation, the role of the hospital changes with the innovation process. Clinicians in hospitals can help BME enterprises find innovation direction [4]. They also can assist BME enterprises in testing prototypes or commenting on the availability of BME products [5]. As one of the consumers, hospitals can be the first to use new medical devices and promote them to suitable patients. They encourage innovation in the BME industry [6] because they can improve their medical technology innovation ability from the development of medical devices—they can even develop new medical devices themselves [7]. At present, almost all high-level public hospitals in China have established partnerships with universities and research institutions. As affiliated hospitals of universities and research institutions, they also undertake the work of medical personnel training and scientific research while serving patients. It is worth noting that almost all the hospitals involved in BME collaborative innovation are public hospitals, while private hospitals are seldom involved.

According to the data provided by the American Hospital Association (AHA) website, among more than 5500 hospitals in the United States, public hospitals account for only 15% of the total number of hospitals in the United States, while private hospitals account for 85%, of which non-profit private hospitals account for 69% and for-profit private hospitals account for 16%. At present, the hospitals with the largest scale, the best facilities and the highest medical level all over the United States are non-profit private hospitals. Cooperation between medical device manufacturers and private hospitals is very common. The number of medical institutions in China is considerable. According to the China Health Statistics Yearbook 2019, by the end of 2018, the number of public hospitals in China was 12,032, while the number of private hospitals was 20,977, almost double that of public hospitals. The number of private hospitals is huge and continues to grow. Although China has a large number of private hospitals, few have independent biomedical engineering departments. According to the World Health Organization published data in 2017 [8], the total number of hospitals in China with biomedical engineering units is 5472, while the number in the USA is 5000. It is not difficult to find that most private hospitals in China do not have their own biomedical engineering units. Due to the lack of biomedical engineering units, BME tends to choose public hospitals when choosing cooperative hospitals.

Moreover, in China, some doctors in public hospitals must treat very high numbers of patients every day. The heavy treatment/surgery workload of the doctors in public hospitals makes it difficult for them to find time for BME innovation. According to the statistical bulletin on the development of health and health services in China in 2018 provided by the Chinese government, the number of patients treated in private hospitals in 2018 was 530 million person-time, while the number of patients treated in public hospitals was 3.05 billion person-time. The average daily number of patients diagnosed and treated by each doctor in public hospitals was 7.5, while that in private hospitals was 5.0. In the United States, private hospitals cover 58% of the U.S. population. In contrast, in China, doctors in private hospitals can have more time to participate in BME innovation than those in public hospitals because of their lower workload. For BME industry innovation in China, private hospitals have great potential to be tapped.

Nowadays, in China, the medical resources and human resources of the vast majority of private hospitals have not been utilized in the innovation of the BME industry. We searched the patent database on the website of the Chinese National Intellectual Property Administration, PRC, and found that almost all the innovation output of biomedical engineering created by hospitals comes from public hospitals. Why do private hospitals with more time for BME innovation rarely participate in collaborative innovation in the BME industry? What factors affect private hospitals’ participation in BME collaborative innovation compared with public hospitals? How do these factors work? Although the existing literature has analyzed the advantages and effects of clinicians’ participation in BME innovation [3,6,7], the research field on the factors influencing private hospitals’ participation in BME collaborative innovation is still sparse. Thus, from the aspects of the novelty and the practicability of innovation projects, the innovation ability of partners and the reputation of private hospitals, this paper attempts to explore how each factor affects the collaborative innovation among private hospitals, public hospitals, BME enterprises, universities and research institutes by using the method of evolutionary game theory and stakeholder theory so as to answer the above questions.

This study can provide a theoretical basis and reference for the small and medium-sized Chinese BME enterprises that do not have independent R & D capabilities in choosing cooperative innovation partners and also can provide theoretical support for the development of the collaborative innovation network of China’s BME industry.

## 2. Literature Review

The development of industrial science and technology has promoted the progress of BME. BME is a discipline that combines engineering and medicine to solve problems in life science research and medical diagnosis and treatment [9]. With the continuous development of engineering technology and the discovery of new materials, the variety of BME products is becoming increasingly rich. For example, the emergence of artificial intelligence is helpful in the diagnosis of patients’ diseases, thus reducing the working pressure of doctors [10]. Reina et al. [11], Ghosal et al. [12] and Chung et al. [13] described the applications and challenges of graphene materials in medicine. Koydemir et al. pointed out that the emergence of smart phones has accelerated the innovation of biomedical instruments [14].

There is a large amount of existing literature that focuses on the innovation of the BME industry from different perspectives. Some scholars paid attention to the innovative business model of the BME industry. Guzzo, D. et al. took the application of coalbed methane in the medical device industry as an example to discuss the sustainable recycling business model of the medical device industry [15]. Maresova, P. et al. provided an analysis and summary of current research in the field of medical device development methodology through a literature review [16]. Some scholars also discussed the impact of medical policies on the innovation of the BME industry. Kesavan, P. et al. discussed the impact of healthcare reform on biomedical engineering technology innovation [17]. The impact of medical resources and environment on BME industry innovation has also attracted the attention of scholars. Richards-Kortum R. and Oden M. pointed out that developers in the BME industry also need to consider the medical resource environment of different regions when designing products [18]. They believe that designers of BME products often take for granted the infrastructure that supports safe use and effective distribution when designing medical equipment. However, these BME products often fail to work in low-resource areas, such as some parts of Africa. In order to achieve the governance objectives of clinical risk management and provide guidance for the formulation of health policies, Health Technology Assessment (HTA) was proposed [19]. However, due to its complexity of critical procedures of the HTA algorithm, HTA is often difficult to apply in practice. Improta G. et al. used analytic hierarchy process (AHP) to try to solve this problem [20]. Pecchia L. and Craven M.P. analyzed the limitations of the widely used Early Stage Health Technology Assessment method in the early evaluation of biomedical equipment and proposed two early evaluation methods for innovative technologies in the BME industry [21].

The innovation of the BME industry needs the coordination of multiple organizations [22]. Collaborative innovation is a coupling process [23]. It is not only the interaction of knowledge, technology, information and resources among different subjects but the integration of resources, decision-making and innovation [24]. A collaborative innovation network with a benign cooperation mechanism can help to promote the development of industrial innovation [25]. Knowledge transfer from members of the collaborative innovation network may enhance the capacity of industries for innovation [26,27]. Some scholars have studied the cooperation network of the BME industry. Based on the configuration theory, Pullon et al. analyzed the new product development cooperation network of small and medium-sized enterprises in the medical device industry and discussed the influence of the network characteristics such as goal complementarity, resource complementarity, fairness trust, reliability trust and network position strength on innovation performance [28]. They pointed out that the interaction of network characteristics is of crucial importance for high innovation performance in the BME industry [29]. Some scholars have analyzed the innovation methods of the BME industry. For example, Beswick et al. analyzed how innovative methods are applied in the field of biophotonics [30].

For the BME industry, as one of the most high-tech industries, it is undeniable that its innovation needs human and financial resources. However, the development of the BME industry in China is still in its infancy. It is difficult for the majority of Chinese BME enterprises to have enough resources for independent innovation. As a new paradigm of science and technology innovation, collaborative innovation can promote enterprises, universities and research institutions to fully develop their own ability, integrate complementary resources and realize the complementary advantages of all parties [2,31]. Therefore, collaborative innovation is a more suitable way for most of China’s BME enterprises to develop at this stage.

According to the theory of collaborative innovation, the main bodies of collaborative innovation are enterprises, governments, knowledge production institutions and intermediary agencies. Due to the particularity of the BME industry, hospitals should also be considered as one of the main bodies of the BME collaborative innovation network. The innovation of the BME industry is inseparable from the cooperation with hospitals. Hospitals, one of the consumers of BME enterprises, can help enterprises determine clinical needs and test prototypes or comment on the availability of products [5]. Sack et al. described the design of pediatric equipment through collaboration between a children’s hospital and two engineering schools [32]. They believed that cooperation between the colleges and the children’s hospital could play an effective role in the prototype development of new pediatric medical equipment. Furthermore, changes in hospital decisions also affect the innovation of the biomedical engineering service industry. With the gradual optimization of the patient treatment process in hospitals, supporting medical service industries have emerged, such as the development of online hospitals and mobile medical software. Some scholars conducted research on the optimization of patient treatment procedures in hospitals. For example, Ricciardi, C. et al. [33] and Giovanni, I. et al. [34,35] applied the Six Sigma method to the patient hospital service, and their research results proved that the service mode combined with the Six Sigma method can improve the efficiency of health management personnel for patients.

Most extant literature about BME collaborative innovation focused on the cultivation of innovative talents in BME. Kotche et al. discussed the impact of clinical immersion programs on the cultivation of BME undergraduates [36]. They found that these programs were impactful for both their career interests and ability to find their first jobs. Similarly, Hodgson et al. [37] and Douglas [38] studied the impact of cooperative projects on the cultivation of innovative talent in BME. They believed that cooperative projects were beneficial to the cultivation of these innovative talents. However, after analyzing the existing literature, it can be found that few studies focus on the role of hospitals in the innovation of the BME industry.

In particular, the role of private hospitals has hardly been mentioned.

Evolutionary game theory has been a powerful method for demonstrating the evolution of cooperation which is widely used to analyze cooperative relationships between stakeholders [39]. Different from static game, the evolutionary game model considers the rational degree of stakeholders [40]. Kai et al. analyzed the low-carbon strategic choice of supply chain enterprises by evolutionary game theory [41]. Lan et al. used evolutionary game theory to investigate the various factors which influence the strategy selection of the parties in school sports injury accidents [42]. Chen et al. discussed the interaction and influencing factors among enterprise pollution control, government supervision and public participation [43]. Moreover, evolutionary game theory is also used in the treatment and diagnosis of diseases. Liao et al. [44], Malekian et al. [45] and Orlando et al. [46] used evolutionary game theory combined with engineering technology in the diagnosis and treatment of cancer. Some scholars also used evolutionary game theory to explore the feasibility of the spread and development of new BME technology. For instance, Chen et al. established an evolutionary game model for exploring the diffusion strategies of mobile health promotion [47].

This paper attempts to use evolutionary game theory to explore the impact of novelty, practicability, risk of BME collaborative innovation project, reputation and innovation ability of partners on the collaborative innovation of BME enterprises, universities, scientific research institutes, public hospitals, private hospitals and government. A tripartite game model including BME enterprises, universities, scientific research institutes, public hospitals, private hospitals and government is established to analyze how these factors affect their cooperation. The remainder of the paper is organized as follows. Section 3 presents some assumptions, the model framework and stability analysis. The following section, Section 4, presents the simulation experiments and discussion of the results. Finally, concluding remarks and some suggestions are given in Section 5.

## 3. Model

Organizations can learn and innovate from stakeholder relationships [48]. When organizations cooperate with each other, knowledge flow among partners will produce a knowledge spillover effect [49]. Knowledge spillover among organizations makes new technology and knowledge spread freely and produce a synergistic effect. Innovation partners reduce innovation cost, disperse innovation risk and realize scale benefit through innovation resource sharing.

Three participants in BME industry innovation are considered in this model. The technological innovation in the BME industry is usually proposed by public hospitals, enterprises, universities and research institutions because they have more sufficient resources than private hospitals for innovation. Therefore, the organizations which have the innovation needs and begin to initiate innovation projects are defined as innovation project initiators in this model, such as public hospitals, enterprises, universities and research institutions. To facilitate expression, the innovation project initiators (IPI) are marked as player IPI, and the private hospitals participating in innovation are marked as player PRH. Another participant is the government. According to stakeholder theory and evolutionary game theory, the first assumption can be proposed below as the basis of the model.

**Assumption** **1.** *All of the participants in this model are boundedly rational. Each of them has two strategies to choose from.*

The initiators of BME innovation projects who can innovate independently often need to have high innovation ability and strong financial support to bear all the innovation risks and costs independently. However, at present, most enterprises, hospitals, universities and research institutions in China do not have these strengths. Hence, player IPI that does not have high innovation ability and strong financial support has two strategies to choose from in this model when they want to cooperate with hospitals. One is to cooperate with other public hospitals (S1), and the other is to cooperate with private hospitals (S2).

Player PRH also has two strategies: participate in innovation or not. When player PRH takes the non-cooperation strategy, player IPI has to cooperate with public hospitals. The parameters *R* (*R* > 0) and *C* (*C* > 0) respectively represent the total revenue and total cost of all partners choosing the collaborative innovation strategy. $\varphi_{\mathrm{PU}}^{S1}$ and $\varphi_{\mathrm{PR}}^{\mathrm{S2}}$ represent the proportion of public hospitals and private hospitals in *R*, while $\xi_{\mathrm{PU}}^{\mathrm{S1}}$ and $\xi_{\mathrm{PR}}^{\mathrm{S2}}$ are the proportion of public hospitals and private hospitals in *C*, respectively.

Most of the public hospitals are supported by the government, so they usually have good credit for their innovative partners. Private hospital refers to the non-governmental public hospitals with a private nature. Most of the private hospitals are funded by profit-making individuals or groups. Only a few private hospitals are non-profit organizations and enjoy government subsidies. Hence, for their innovative partners, it is difficult to guarantee the credibility of private hospitals.

The government’s strategy is to supervise the cooperative innovation of private hospitals or not. Supervising the cooperative innovation of private hospitals can reduce the innovation risks entailed by private hospitals’ participation in collaborative innovation. The reduction is marked as *RCRD*. The supervision from the government means that the government must pay the cost of supervision (*CS*). When the innovation project is successful, the income of innovation project initiator and private hospital increases. At the same time, the government will obtain more taxes. This tax increment is recorded as *GRG*. *GRG*, *CS* > 0.

**Assumption** **2.** *The novelty and practicability of the innovation project and the collaborative innovation ability and credit of collaborators affect this project’s total revenue and total cost.*

In this paper, two categories of factors are concerned which affect *R* and *C*. One is the characteristics of the innovation project itself, including the novelty and practicability. Novelty represents the degree of innovation of the innovation project—that is, the proportion of new methods, new technologies or new materials in the innovation project. The parameter *n* (0 ≤ *n* ≤ 1) is used to present the novelty. The higher the parameters *n* is, the higher the total revenue, total cost and total profit of the project is, and the higher the risk of the project’s innovation is. When *n* = 1, it means the innovation project is a breakthrough innovation. The practicability of the innovation project, which is marked as *u* (0 ≤ *u* ≤ 1) in this model, represents the extent to which the project can be used in existing markets. The higher the parameters *u* is, the higher *R* is.

The other category is the characteristics of collaborators, including the collaborative innovation ability (*CIA*) and credit of collaborators. The collaborative innovation ability includes the technological innovation ability and cooperation ability of the cooperators. When the collaborators have strong innovation ability, they can more easily develop new methods and technologies or find new materials than those collaborators with lower capabilities. When they have strong cooperation ability, they can reduce the cost of cooperation and increase cooperation revenue. The parameter *CIA* (0 ≤ *CIA* ≤ 1) is used to indicate the collaborative innovation capabilities of collaborators. The collaborative innovation abilities of Player IPI, Player PRHU and public hospitals are marked as *CIA*_IPI_, *CIA*_PRH_ and *CIA*_PUH_, respectively.

In this model, the credit of private hospitals is considered. The parameter *Cr* is used to represent the credit of private hospitals, −1 ≤ *Cr* ≤ 1. The larger the *Cr* is, the higher the credit of private hospitals is, and the lower the cost of innovation risk cost borne by the partner. It should be noted that the lower the *Cr*, the higher the cost of innovation risk cost borne by the partner.

As one of the most high-tech industries, the BME industry has a high risk of failure in innovation. The risk cost (*RC*) of the innovation project is used to represent the costs that all participants must pay for innovation failure. When Player IPI chooses strategy S1 or S2, $\gamma_{\mathrm{PUH}}^{S1}$ and $\gamma_{\mathrm{PRH}}^{S2}$ represent the proportion of public hospitals and private hospitals in *RC*, respectively.

To show the parameters more clearly, all parameters and their definitions are presented in Table 3.

**Assumption** **3.** *The effect of each influencing factor increases with the increase in its absolute value.*

In order to approximate the impact of different factors on revenue, cost and risk, the following equation is used to represent their effects.

(1) $$Z=\left\{ \begin{array}{l} 1-\left(1-t\right)e^{t},t\in [0,1]\\ -[1-\left(1-|t|\right)e^{\left|t\right|}],t\in [-1,0) \end{array}\right.$$

where *t* is the influencing factor and *Z* is the effect degree of *t*. The larger the absolute value of *t* is, the more obvious the effect. According to the definition of factors in Assumption 2, the revenues, costs and risks of innovation projects under different strategies can be obtained, as shown in Table 4.

The parameters *m*_0–5_, *l*_0–4_, and *k*_0–5_ in Table 4 are coefficients and all non-negative. Their values are determined by the innovation project itself.

The payoff of each player under different strategies are shown in following Table 5.

The probability of Player IPI adopting strategy S2 is set to *x*. Similarly, the probability of Player PRH taking the collaborative innovation strategy and the probability of the government taking the supervision strategy are set to *y* and *z*, respectively, *x*, *y*, *z* ∈ [0,1]. The expected payoff of each player’s strategy is marked as $\psi_{\mathrm{player}}^{\mathrm{Strategy}}$.

(8) $$\psi_{\mathrm{IPI}}^{S1}=(1-\varphi_{\mathrm{PU}}^{S1})R_{1}-(1-\xi_{\mathrm{PU}}^{S1})C_{1}-(1-\gamma_{\mathrm{PU}}^{S1})RC_{1}$$

(9) $$\begin{array}{cc} \psi_{\mathrm{IPI}}^{S2} & =yz[(1-\varphi_{\mathrm{PR}}^{S2})R_{2}-(1-\xi_{\mathrm{PR}}^{S2})C_{2}-(1-\gamma_{\mathrm{PR}}^{S2})(RC_{2}-RCRD)]+y(1-z)[(1-\varphi_{\mathrm{PR}}^{S2})R_{2}-(1-\xi_{\mathrm{PR}}^{S2})C_{2}-(1-\\ & \gamma_{\mathrm{PR}}^{S2})RC_{2}]+(1-y)[(1-\varphi_{\mathrm{PU}}^{S1})R_{1}-(1-\xi_{\mathrm{PU}}^{S1})C_{1}-(1-\gamma_{\mathrm{PU}}^{S1})RC_{1}]\\ & =yz(1-\gamma_{\mathrm{PR}}^{S2})RCRD+y[(1-\varphi_{\mathrm{PR}}^{S2})R_{2}-(1-\xi_{\mathrm{PR}}^{S2})C_{2}-(1-\gamma_{\mathrm{PR}}^{S2})RC_{2}-(1-\varphi_{\mathrm{PU}}^{S1})R_{1}+(1-\xi_{\mathrm{PU}}^{S1})C_{1}+\\ & (1-\gamma_{\mathrm{PU}}^{S1})RC_{1}]+(1-\varphi_{\mathrm{PU}}^{S1})R_{1}-(1-\xi_{\mathrm{PU}}^{S1})C_{1}-(1-\gamma_{\mathrm{PU}}^{S1})RC_{1} \end{array}$$

(10) $$\psi_{\mathrm{PRH}}^{y}=xz[\varphi_{\mathrm{PR}}^{S2}R_{2}-\xi_{\mathrm{PR}}^{S2}C_{2}-\gamma_{\mathrm{PR}}^{S2}(RC_{2}-RCRD)]+x(1-z)[\varphi_{\mathrm{PR}}^{S2}R_{2}-\xi_{\mathrm{PR}}^{S2}C_{2}-\gamma_{\mathrm{PR}}^{S2}RC_{2}]$$

(11) $$\psi_{\mathrm{PRH}}^{1-y}=0$$

(12) $$\psi_{G}^{z}=xyGRG-CS$$

(13) $$\psi_{G}^{1-z}=0$$

Based on Table 5, average expected payoff of each player is displayed as follows:

(14) $$\bar{\psi_{\mathrm{IPI}}}=x\psi_{\mathrm{IPI}}^{S2}+(1-x)\psi_{\mathrm{IPI}}^{S1}$$

(15) $$\bar{\psi_{\mathrm{PRH}}}=y\psi_{\mathrm{PRH}}^{y}+(1-y)\psi_{\mathrm{PRH}}^{1-y}$$

(16) $$\bar{\psi_{G}}=z\psi_{G}^{z}+(1-z)\psi_{G}^{1-z}$$

Evolutionary game theory holds that players cannot find the optimal strategy from the beginning because they are bounded rational. They need to learn, improve and imitate in the process of the game. Finally, all players will approach a stable strategy, which is called evolutionary stable strategy (ESS). Replication dynamics is actually a dynamic differential equation that describes the rate at which the frequency of strategies used in a population changes over time [50]. Then, the replication dynamic system of this model can be obtained as follows:

(17) $$\begin{array}{cc} \frac{dx}{dt} & =x(\psi_{\mathrm{IPI}}^{S2}-\bar{\psi_{\mathrm{IPI}}})=x(1-x)(\psi_{\mathrm{IPI}}^{S2}-\psi_{\mathrm{IPI}}^{S1})\\ & =x(1-x)\{yz[(1-\varphi_{\mathrm{PR}}^{S2})R_{2}-(1-\xi_{\mathrm{PR}}^{S2})C_{2}-(1-\gamma_{\mathrm{PR}}^{S2})(RC_{2}-RCRD)]+\\ & y(1-z)[(1-\varphi_{\mathrm{PR}}^{S2})R_{2}-(1-\xi_{\mathrm{PR}}^{S2})C_{2}-(1-\gamma_{\mathrm{PR}}^{S2})RC_{2}]-\\ & y[(1-\varphi_{\mathrm{PU}}^{S1})R_{1}-(1-\xi_{\mathrm{PU}}^{S1})C_{1}-(1-\gamma_{\mathrm{PU}}^{S1})RC_{1}]\}\\ & =x(1-x)\{yz(1-\gamma_{\mathrm{PR}}^{S2})RCRD+y[(1-\varphi_{\mathrm{PR}}^{S2})R_{2}-(1-\xi_{\mathrm{PR}}^{S2})C_{2}-(1-\gamma_{\mathrm{PR}}^{S2})RC_{2}\\ & -(1-\varphi_{\mathrm{PU}}^{S1})R_{1}+(1-\xi_{\mathrm{PU}}^{S1})C_{1}+(1-\gamma_{\mathrm{PU}}^{S1})RC_{1}]\} \end{array}$$

(18) $$\begin{array}{cc} \frac{dy}{dt} & =y(\psi_{\mathrm{PRH}}^{y}-\bar{\psi_{\mathrm{PRH}}})=y(1-y)\psi_{\mathrm{PRH}}^{y}\\ & =y(1-y)\{xz[\varphi_{\mathrm{PR}}^{S2}R_{2}-\xi_{\mathrm{PR}}^{S2}C_{2}-\gamma_{\mathrm{PR}}^{S2}(RC_{2}-RCRD)]+x(1-z)(\varphi_{\mathrm{PR}}^{S2}R_{2}-\xi_{\mathrm{PR}}^{S2}C_{2}-\gamma_{\mathrm{PR}}^{S2}RC_{2})\}\\ & =y(1-y)[xz\gamma_{\mathrm{PR}}^{S2}RCRD+x(\varphi_{\mathrm{PR}}^{S2}R_{2}-\xi_{\mathrm{PR}}^{S2}C_{2}-\gamma_{\mathrm{PR}}^{S2}RC_{2})] \end{array}$$

(19) $$\begin{array}{cc} \frac{dz}{dt} & =z(\psi_{G}^{z}-\bar{\psi_{G}})=z(1-z)\psi_{G}^{z}\\ & =z(1-z)(xyGRG-CS) \end{array}$$

The system above is a non-linear system that has a continuous first derivative. Based on Lyapunov’s stability first theorem [51], the Jacobian matrix of this system can be obtained as Equation (20).

(20) $$J=\left(\begin{array}{ccc} a_{11} & a_{12} & a_{13}\\ a_{21} & a_{22} & a_{23}\\ a_{31} & a_{32} & a_{33} \end{array}\right)$$

where

(21) $$\begin{array}{cc} a_{11} & =(1-2x)\{yz(1-\gamma_{\mathrm{PR}}^{S2})RCRD+y[(1-\varphi_{\mathrm{PR}}^{S2})R_{2}-(1-\xi_{\mathrm{PR}}^{S2})C_{2}-(1-\gamma_{\mathrm{PR}}^{S2})RC_{2}\\ & -(1-\varphi_{\mathrm{PU}}^{S1})R_{1}+(1-\xi_{\mathrm{PU}}^{S1})C_{1}+(1-\gamma_{\mathrm{PU}}^{S1})RC_{1}]\} \end{array}$$

(22) $$a_{12}=x(1-x)\{z(1-\gamma_{\mathrm{PR}}^{S2})RCRD+[(1-\varphi_{\mathrm{PR}}^{S2})R_{2}-(1-\xi_{\mathrm{PR}}^{S2})C_{2}-(1-\gamma_{\mathrm{PR}}^{S2})RC_{2}\}$$

(23) $$a_{13}=x(1-x)[y(1-\gamma_{\mathrm{PR}}^{S2})RCRD]$$

(24) $$a_{21}=y(1-y)[z\gamma_{\mathrm{PR}}^{S2}RCRD+(\varphi_{\mathrm{PR}}^{S2}R_{2}-\xi_{\mathrm{PR}}^{S2}C_{2}-\gamma_{\mathrm{PR}}^{S2}RC_{2})]$$

(25) $$a_{22}=(1-2y)[xz\gamma_{\mathrm{PR}}^{S2}RCRD+x(\varphi_{\mathrm{PR}}^{S2}R_{2}-\xi_{\mathrm{PR}}^{S2}C_{2}-\gamma_{\mathrm{PR}}^{S2}RC_{2})]$$

(26) $$a_{23}=y(1-y)[x\gamma_{\mathrm{PR}}^{S2}RCRD]$$

(27) $$a_{31}=z(1-z)(yGRG)$$

(28) $$a_{32}=z(1-z)(xGRG)$$

(29) $$a_{33}=(1-2z)(xyGRG-CS)$$

When *x*, *y* and *z* ∈ [0,1] ensure that the replication dynamics in Equations (17)–(19) are equal to 0, the point (*x*,*y*,*z*) is the equilibrium point. Obviously, the points (0,0,0) (1,0,0), (0,1,0), (0,0,1), (1,0,1), (1,1,0), (0,1,1), (1,1,1) are equilibrium in this evolutionary game system. Furthermore, when 0 ≤ *x*’, *y*’, *z*’ ≤ 1 and they can make Equations (30)–(32) work, the point (*x*’,*y*’,*z*’) is also an equilibrium point.

(30) $$z(1-\gamma_{\mathrm{PR}}^{S2})RCRD+[(1-\varphi_{\mathrm{PR}}^{S2})R_{2}-(1-\xi_{\mathrm{PR}}^{S2})C_{2}-(1-\gamma_{\mathrm{PR}}^{S2})RC_{2}-(1-\varphi_{\mathrm{PU}}^{S1})R_{1}+(1-\xi_{\mathrm{PU}}^{S1})C_{1}+(1-\gamma_{\mathrm{PU}}^{S1})RC_{1}]=0$$

(31) $$z\gamma_{\mathrm{PR}}^{S2}RCRD+(\varphi_{\mathrm{PR}}^{S2}R_{2}-\xi_{\mathrm{PR}}^{S2}C_{2}-\gamma_{\mathrm{PR}}^{S2}RC_{2})=0$$

(32) xyGRG−CS=0

According to modern control theory, the stability of each equilibrium point can be determined by distinguishing the positive and negative of *J*’s eigenvalues *λ*i (i = 1,2,3). When all *λ*i of the equilibrium point (*x*,*y*,*z*) are negative, this equilibrium point is asymptotically stable and the strategy represented by this equilibrium point is ESS. If the eigenvalue *λ* of point (*x*,*y*,*z*) is 0, then the point may be stable, but it must not be asymptotically stable. Therefore, the asymptotic stability of the equilibrium point can be verified by the non-negativity of its eigenvalues. It is worth noting that the elements on the main diagonal of the Jacobian matrix of point (*x*’, *y*’, *z*’) are all 0. This means that the sum of eigenvalues of the matrix is 0 and it is impossible for all eigenvalues of this point to be negative at the same time. Therefore, point (*x*’, *y*’, *z*’) is not an asymptotically stable point.

All of the equilibrium point’s asymptotically stable conditions and stability are shown in Table 6. In this model, there are three points that may be asymptotically stable, namely point (1,0,0), (1,1,0) and (1,1,1).

## 4. Simulation and Discussion

In order to intuitively analyze the influence of different parameters on the result of strategy evolution, this paper simulates the strategy selection process of BME enterprises, public hospitals, private hospitals, universities and research institutions in different initial states using MATLAB 2019 software. Three sets of parameters are randomly set to represent the evolutionary game system in three different stable states. On this basis, the influence of these variables on the stability of evolutionary game is discussed by changing the values of novelty, practicality, innovation ability and credit. The values of all parameters in different scenarios are set as following Table 7.

**Figure 1:**
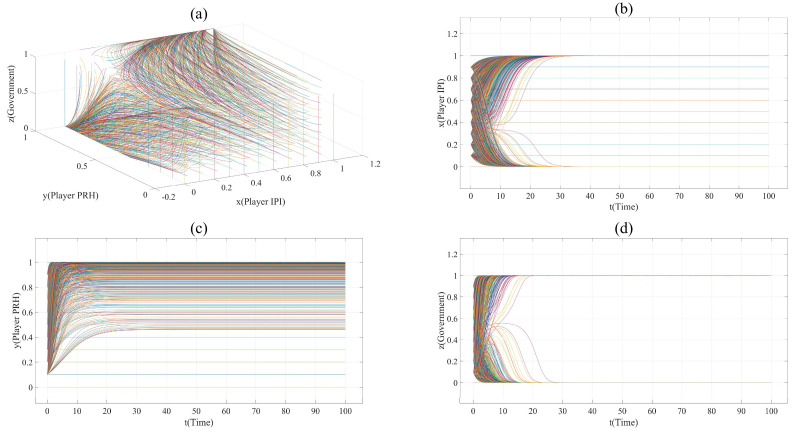
(**a**). All evolution paths in Scenario 1. (**b**). Evolution paths of Player IPI in Scenario 1. (**c**). Evolution paths of Player PRH in Scenario 1. (**d**). Evolution paths of government in Scenario 1.

According to Table 7, the value of *x*, *y* and *z* in Scenario 1 are taken every 0.1 from 0 to 1, time *t* (unit time) from 0 to 100. All the evolution paths in Scenario 1 can be displayed in Figure 1a. Due to the selection method of *x*, *y* and *z*, there are 1331 curves in Figure 1a. Therefore, it is impossible to mark each curve with a legend. In the following figures, each curve is given different colors to represent their evolution more clearly.

The assignment of each parameter in Scenario 1 describes such a situation: Player IPI and Player PRH evenly distribute the revenue cost and risk of innovation ($\varphi_{\mathrm{PU}}^{S1}$ = $\xi_{\mathrm{PU}}^{S1}$ = $\gamma_{\mathrm{PU}}^{S1}$ = $\varphi_{\mathrm{PR}}^{S2}$ = $\xi_{\mathrm{PR}}^{S2}$ = $\gamma_{\mathrm{PU}}^{S2}$ = 0.5); the impact of innovation novelty on risk and cost is moderate, and the impact of the innovation project’s practicability and novelty on revenue is obvious (*m*1 = 20, *l*1 = 4.5, *k*1 = 3, *m*2 = 10); when the government does not supervise, the profit of Player IPI cooperating with public hospitals is higher than that with private hospitals (e.g., (1 − $\varphi_{\mathrm{PU}}^{S1}$)*R*_1_ − (1 − $\xi_{\mathrm{PU}}^{S1}$)*C*_1_ − (1 − $\gamma_{\mathrm{PU}}^{S1}$) *RC*_1_ > (1 − $\varphi_{\mathrm{PR}}^{S2}$)*R*_2_ − (1 − $\xi_{\mathrm{PR}}^{S2}$)*C*_2_ − (1 − $\gamma_{\mathrm{PU}}^{S2}$)*RC*_2_); the credit of private hospitals is not high (*Cr* = 0.2), and their collaborative innovation ability is not as good as that of public hospitals (e.g., *CIA*_PRH_ < *CIA*_PUH_). When Player IPI and Player PRH cooperate, the government can benefit from the supervision strategy (*GRG* − *CS* > 0), and the innovation risk reduction brought about by the government’s supervision can make the payoff of Player IPI taking strategy S2 larger (e.g., 0 < (1 − $\varphi_{\mathrm{PU}}^{S1}$)*R*_1_ − (1 − $\xi_{\mathrm{PU}}^{S1}$)*C*_1_ − (1 − $\gamma_{\mathrm{PU}}^{S1}$) *RC*_1_ < (1 − $\varphi_{\mathrm{PR}}^{S2}$)*R*_2_ − (1 − $\xi_{\mathrm{PR}}^{S2}$)*C*_2_ − (1 − $\gamma_{\mathrm{PU}}^{S2}$)*(RC*_2_ + *RCRD*) ).

Figure 1 shows the evolution paths in this scenario. It can be found that this system has an asymptotically stable point (1,1,1). This indicates that, under the supervision of the government, collaborative innovation between private hospitals and innovation project initiators is the finally stable strategy. However, it is worth noting that some vertical lines appear in plane (*x* = 0, 0 ≤ *y* ≤ 1, 0≤ *z* ≤1) and plane (*y* = 0, 0 ≤ *x* ≤ 1, 0 ≤ *z* ≤1) in Figure 1a, and some horizontal lines appear in Figure 1b,c. This is because when *x* = 0 or *y* = 0, the change in Player IPI’s and Player PRH’s strategy will not affect each other’s payoff. This shows that as long as the initiator of the innovation project chooses to cooperate with public hospitals, or private hospitals choose the non-participation strategy, the strategy change of both sides will not change each other’s strategy, and the government will choose the non-supervision strategy. Therefore, the points in the line (*x* = 0, 0 ≤ *y* ≤ 1) and the line (*y* = 0, 0 ≤ *x* ≤ 1) are stable, but not asymptotically stable.

**Figure 2:**
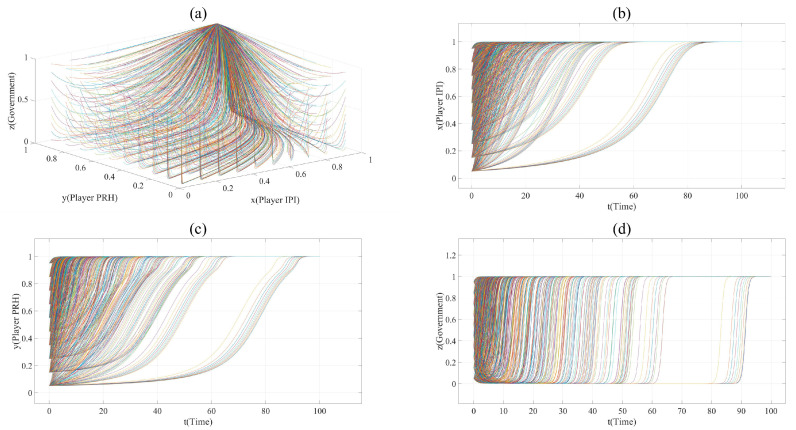
(**a**). All evolution paths in Scenario 2. (**b**). Evolution paths of Player IPI in Scenario 2. (**c**). Evolution paths of Player PRH in Scenario 2. (**d**). Evolution paths of government in Scenario 2.

On the basis of Scenario 1, the proportion of revenue, cost and risk has changed in Scenario 2. Player IPI occupies more profits and lower risk (e.g., $\varphi_{\mathrm{PR}}^{S2}$ = $\xi_{\mathrm{PR}}^{S2}$ = 0.3, $\gamma_{\mathrm{PU}}^{S2}$ = 0.6). In order to show the evolution trend of each point more clearly and reduce the interference caused by *x* = 0 or *y* = 0 to the image, the value of *x* and *y* in Scenario 2 is taken every 0.1, from 0.05 to 1. The evolution paths of Scenario 2 are shown in Figure 2. The point (1,1,1) is the only asymptotically stability point in Scenario 2. This shows that, compared with Scenario 1, the private hospitals in Scenario 2 sacrifice part of their profits and undertake more innovation risks, but they gain more opportunities to participate in the collaborative innovation of the BME industry.

In order to observe the effect of each factor more clearly, the evolution curve of point (*x* = 0.2, *y* = 0.3, *z* = 0.8) is selected. On the basis of the parameter set in Scenario 2, the impact of *n*, *u*, *CIA* and *Cr* can be shown in following figures by changing the values of them.

**Figure 3:**
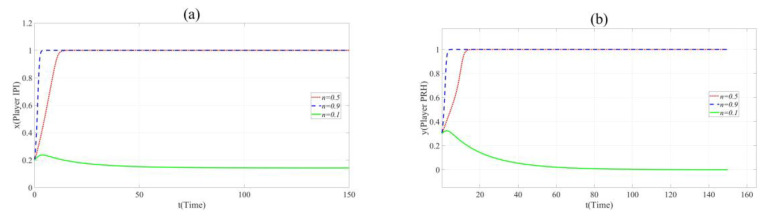
(**a**). Evolution paths of *x* = 0.2 in Scenario 2 when *n* = 0.1, 0.5 and 0.9. (**b**). Evolution paths of *y* = 0.3 in Scenario 2 when *n* = 0.1, 0.5 and 0.9.

According to Figure 3a,b, it can be found that in Scenario 2, with the increase in *n*, the cooperation between Player IPI and Player PRH is promoted. When *n=* 0.1, $\varphi_{\mathrm{PR}}^{S2}$*R*_2_ − $\xi_{\mathrm{PR}}^{S2}$*C*_2_ − $\gamma_{\mathrm{PU}}^{S2}$*RC*_2_ < 0, Player PRH will not cooperate with Player IPI. When *n* = 0.9, the time *t* for both sides to choose the cooperative innovation strategy is significantly reduced. This means that when the novelty of the project cannot significantly affect the innovation risk, the novelty promotes collaborative innovation.

Nevertheless, it should be noted that *k*_1_= 3 in Scenario 1 and Scenario 2 while *k*_1_= 16 in Scenario 3. This means that the impact of novelty on the innovation risk is not obvious in Scenario 1 and Scenario 2 but the change in innovation novelty will greatly affect the risk of the collaborative innovation project in Scenario 3. Similarly, the evolution curve of point (*x* = 0.2, *y* = 0.3, *z* = 0.8) is selected in Scenario 3, and the evolutionary path figures are as follows.

**Figure 4:**
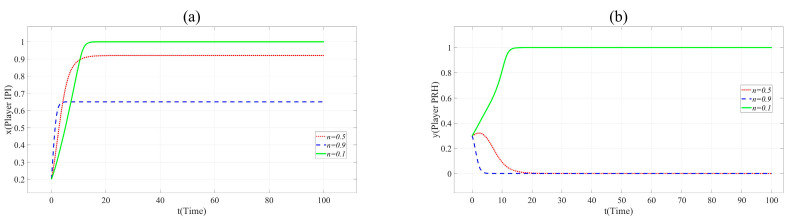
(**a**). Evolution paths of *x* = 0.2 in Scenario 3 when *n* = 0.1, 0.5 and 0.9. (**b**). Evolution paths of *y* = 0.3 in Scenario 3 when *n* = 0.1, 0.5 and 0.9.

According to Figure 4a,b, the increase in *n* inhibited the cooperation between Player PRH and Player IPI. This indicates that when the novelty of the project can bring huge innovation risk, the party undertaking higher innovation risk will reduce the probability of participating in collaborative innovation due to the promotion of novelty.

**Figure 5:**
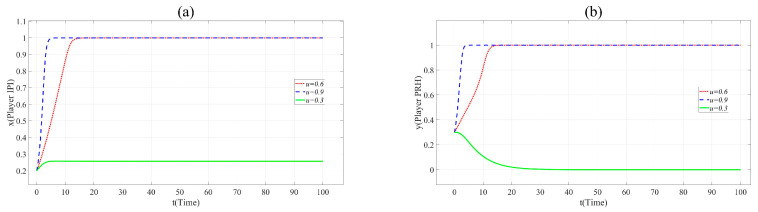
(**a**). Evolution paths of *x* = 0.2 in Scenario 3 when *u* = 0.3, 0.6 and 0.9. (**b**). Evolution paths of *y* = 0.3 in Scenario 3 when *u* = 0.3, 0.6 and 0.9.

**Figure 6:**
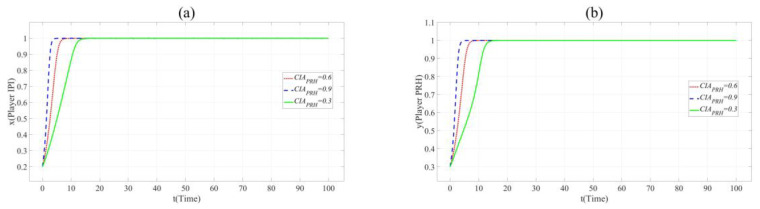
(**a**). Evolution paths of *x* = 0.2 in Scenario 3 when *CIA*_PRH_ = 0.3, 0.6 and 0.9. (**b**). Evolution paths of *y* = 0.3 in Scenario 3 when *CIA*_PRH_ = 0.3, 0.6 and 0.9.

**Figure 7:**
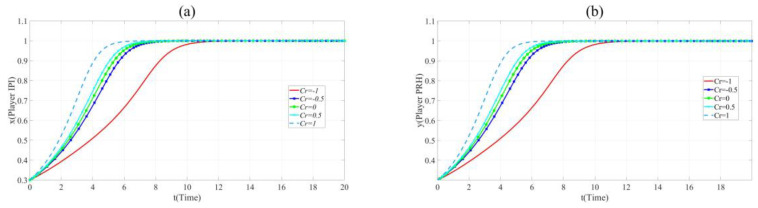
(**a**). Evolution paths of *x* = 0.2 in Scenario 3 when *Cr* = −1, −0.5, 0, 0.5 and 1. (**b**). Evolution paths of *y* = 0.3 in Scenario 3 when *Cr* = −1, −0.5, 0, 0.5 and 1.

Similarly, the influence of the practicality (*u*), the collaborative innovation ability of Player PRH (*CIA*_PRH_) and credit (*Cr*) on collaborative innovation can be shown in Figure 5, Figure 6 and Figure 7.

In Figure 5, with the increase of *u*, *x* and *y* change the direction and speed of evolution. When *u* = 0.9, *x* and *y* eventually evolve to 1, and the evolution speed is faster than that when *u* = 0.6. It means that the practicability of BME innovation project is positively related to the cooperation of partners. Innovation beyond the production capacity of BME industry is often not favored by partners.

Figure 6 shows that the increase of the value of *CIA* accelerates the evolution of *x* and *y* to 1. This shows that private hospitals with strong innovation ability are more popular with the partners.

As shown in Figure 7, the evolution speed of *x* and *y* increases with the increase of *Cr*. This reveals that enhancing the credit of private hospitals contribute to BME collaborative innovation.

Based on the above stability analysis for three different scenarios, the simulation results clearly show the influence of different factors on the cooperation between each player. Compared with Scenario 1 and Scenario 2, it can be found that the stability of the evolutionary game system is affected by the differences in innovation revenue, cost and innovation risk sharing proportion of collaborative innovators. This shows that reasonable allocation of the innovation income, cost and innovation risk of each collaborative innovator can promote the collaborative innovation of the BME industry. The simulation results of Scenario 3 reveal that that the more practical the innovation project, the stronger the collaborative innovation ability of private hospitals, and the higher the credibility of private hospitals, the more conducive to the collaborative innovation of the BME industry. It is worth mentioning that in Scenario 2, the improvement of the innovation project’s novelty shortens the time for collaborative innovators evolving to a stable strategy, thus promoting BME collaborative innovation. Nevertheless, in Scenario 3, the promotion of novelty inhibits collaborative innovators’ cooperation. This shows that when the novelty of the cooperative innovation project can significantly affect the innovation risk, the novelty may not be conducive to collaborative innovation. In short, when the initiators of BME innovation projects choose to cooperate with hospitals, it is necessary to consider not only the actual situation of public hospitals and private hospitals but also the characteristics of the innovation project itself.

## 5. Conclusions

Based on evolutionary game theory and stakeholder theory, this paper constructs a tripartite game model to study the collaborative innovation relationship among BME enterprises, universities, research institutions, public hospitals, private hospitals and the government from the aspects of the novelty and the practicability of innovation projects, the innovation ability of partners and the reputation of private hospitals. Then, it analyzes how these factors affect BME collaborative innovation through the simulation of three scenarios. The results show that (1) the good credit of private hospitals has a positive effect on their participation in collaborative innovation; (2) it is helpful for BME collaborative innovation to enhance the collaborative innovation ability of partners; (3) the novelty of innovation projects has an impact on BME collaborative innovation. The specific impacts depend on the revenue, cost and risk allocation ratio of innovation partners; (4) the higher the practicability of innovation projects, the more conducive to collaborative innovation.

According to the simulation results, the conclusions are obtained as follows. (1) The unreasonable allocation of innovation revenue, cost and innovation risk among partners leads to less participation of private hospitals in collaborative innovation in the BME industry. It is beneficial for the collaborative innovation of BME industry that the cooperators should know their own benefits and costs and reasonably share the innovation risks. (2) The high risk brought by the novelty of the BME industry reduces the enthusiasm of private hospitals to participate in collaborative innovation. This shows that partners are not willing to develop a new BME technology or product because of the high risk of innovation failure. (3) The higher the innovation ability of private hospitals, the more attractive their partners are. The lack of collaborative innovation ability of private hospitals makes it impossible to attract the initiators of BME innovation projects to cooperate with them. (4) The higher the credibility of private hospitals, the more attractive their partners are. The credit of public hospitals is guaranteed by the government, while private hospitals which are mainly for profit often lack credit guarantee. As a result, the BME initiators of BME innovation projects tend to choose public hospitals when they are looking for hospital partners.

Based on the above conclusions, some suggestions are put forward as follows:

(1) The lack of credit has been an important factor restricting the sustainable development of private hospitals in China. Private hospitals should enhance their credit and create a brand of integrity for BME collaborative innovation. In addition, the government should establish a fair and effective credit scoring system for private hospitals, which can provide a reference for the collaborative innovation among BME enterprises, universities, public hospitals and research institutions (2) In order to provide high-level human resources for BME collaborative innovation, private hospitals can improve their doctors’ collaborative innovation ability by attracting talent and joint training. (3) To reduce the impact of the novelty of cooperative innovation projects on cooperation, the partners should actively communicate with each other so as to reasonably distribute the benefits, costs and innovation risks. (4) BME innovators should follow the pace of scientific and technological progress and formulate innovation projects reasonably according to the actual situation of themselves and the industry.

This paper can provide a theoretical basis for future BME innovation research. However, there are some limitations in this study; for example, the influence of geographical factors on cooperation is not considered and the stakeholders of BME industry innovation are not fully defined. Based on this study, the characteristics of the BME collaborative innovation network, how the government establishes a fair and effective credit rating system for private hospitals and how private hospitals effectively improve their collaborative innovation ability will become the next research directions in the future.

## Figures and Tables

**Table 1:** Top 10 export commodities of medical devices in China in 2019.

Top	The Harmonization System Code (HS-Code)	Commodity	Export (Billion Dollars)
1	90191010	Massage apparatus	3.06
2	95069119	Other equipment for exercise and recovery	2.65
3	90318090	Measuring or checking instruments, appliances and machines, nes	2.63
4	90183900	Needles nes, catheters, cannulae and the like	1.48
5	90189099	Medical/veterinary instruments and appliances, nes	1.37
6	90279000	Microtomes; parts and accessories of instruments and apparatus of 90.27	1.20
7	96190011	Diapers and napkins for babies, of any materials	1.01
8	732490	Sanitary ware and parts thereof, i/s, nes, for example bedpans, douche cans	0.99
9	84148090	Other air pumps/ventilation/recycling hoods with fan	0.86
10	30059010	Absorbent cotton, bandage, gauze for retail sale	0.84

**Table 2:** Import and export data of some high-end medical devices in China in 2019.

The Harmonization System Code (HS-Code)	Commodity	Import (Billion Dollars)	Export (Billion Dollars)
90318090	Measuring or checking instruments, appliances and machines, nes	5.34	2.63
90181291	Chromoscope ultrasonic diagnostic equipment	1.09	0.78
90278099	Other instruments and apparatus for 90.27, other than microtomes	2.13	0.75
90221400	X-ray apparatus for medical, surgical or veterinary uses	1.8	0.31
90275000	Other instruments and apparatus using optical radiations, nes	1.84	0.29
90219090	Appliances worn, carried or implanted in the body, nes	1.1	0.22
90181310	Complete equipment of magnetic resonance imaging system (MRI)	0.54	0.18
90189030	Endoscopes	0.99	0.14
90273000	Spectro/spectrophotometers and spectrographs using optical radiations	0.65	0.11
902139	Artificial parts of the body (excluding artificial teeth and dental fittings and artificial joints)	0.65	0.07
90219011	Stents in blood vessels	0.60	0.03
90318031	Apparatus for ultrasonic examinations	0.12	0.01

**Table 3:** The parameters and their definitions.

Parameters	Definition
*R*	The total expected revenue of all partners choosing collaborative innovation strategy
*C*	The total expected cost of all partners choosing collaborative innovation strategy
$\varphi_{\mathrm{PU}}^{S1}$	The proportion of public hospitals in *R*
$\varphi_{\mathrm{PR}}^{S2}$	The proportion of private hospitals in *R*
$\xi_{\mathrm{PU}}^{S1}$	The proportion of public hospitals in *C*
$\xi_{\mathrm{PR}}^{S2}$	The proportion of private hospitals in *C*
*RCRD*	The innovation risks reduction entailed by government’s supervision strategy
*CS*	The government’s cost for supervision strategy
*GRG*	The tax increment of government
*n*	The novelty of the biomedical engineering (BME) innovation project
*u*	The practicability of the BME innovation project
*CIA*	The collaborative innovation ability
*Cr*	The credit of private hospitals
*RC*	The risk cost of the BME innovation project
$\gamma_{\mathrm{PUH}}^{S1}$	The proportion of public hospitals in *RC*
$\gamma_{\mathrm{PRH}}^{S2}$	The proportion of private hospitals in *RC*
*RC*	The risk cost of the innovation project

**Table 4:** The revenues, costs and risks of innovation projects under different strategies.

Strategy	The Revenues, Costs and Risks	
**S1**	$\begin{array}{c} R_{1}=[1-(1-n)e^{n})]\times m_{1}+[1-(1-u)e^{u}]\times m_{2}+[1-(1-CIA_{\mathrm{IPI}})e^{CIA}{}^{{}_{\mathrm{IPI}}})]\times m_{3}\\ \;\;+[1-(1-CIA_{\mathrm{PUH}})e^{CIA}{}^{{}_{\mathrm{PUH}}})]\times m_{4}+m_{0} \end{array}$	(2)
	$C_{1}=[1-(1-n)e^{n})]\times l_{1}-[1-(1-CIA_{\mathrm{IPI}})e^{CIA}{}^{{}_{\mathrm{IPI}}}]\times l_{2}-[1-(1-CIA_{\mathrm{PUH}})e^{CIA}{}^{{}_{\mathrm{PUH}}}]\times l_{3}+l_{0}$	(3)
	$RC_{1}=[1-(1-n)e^{n})]\times k_{1}-[1-(1-CIA_{\mathrm{IPI}})e^{CIA}{}^{{}_{\mathrm{IPI}}}]\times k_{2}-[1-(1-CIA_{\mathrm{PUH}})e^{CIA}{}^{{}_{\mathrm{PUH}}}]\times k_{3}+k_{0}$	(4)
**S2**	$\begin{array}{c} R_{2}=[1-(1-n)e^{n})]\times m_{1}+[1-(1-u)e^{u}]\times m_{2}+[1-(1-CIA_{\mathrm{IPI}})e^{CIA}{}^{{}_{\mathrm{IPI}}})]\times m_{3}\\ \;\;+[1-(1-CIA_{\mathrm{PRH}})e^{CIA}{}^{{}_{\mathrm{PRH}}})]\times m_{5}+m_{0} \end{array}$	(5)
	$C_{2}=[1-(1-n)e^{n})]\times l_{1}-[1-(1-CIA_{\mathrm{IPI}})e^{CIA}{}^{{}_{\mathrm{IPI}}}]\times l_{2}-[1-(1-CIA_{\mathrm{PRH}})e^{CIA}{}^{{}_{\mathrm{PRH}}}]\times l_{4}+l_{0}$	(6)
	$RC_{2}=\left\{ \begin{array}{l} {}[1-(1-n)e^{n})]\times k_{1}-[1-(1-CIA_{\mathrm{IPI}})e^{CIA}{}^{{}_{\mathrm{IPI}}}]\times k_{2}-[1-(1-CIA_{\mathrm{PRH}})e^{CIA}{}^{{}_{\mathrm{PRH}}}]\times k_{4}\\ -[1-(1-Cr)e^{Cr}]\times k_{5}+k_{0},Cr\in (0,1]\\ {}[1-(1-n)e^{n})]\times k_{1}-[1-(1-CIA_{\mathrm{IPI}})e^{CIA}{}^{{}_{\mathrm{IPI}}}]\times k_{2}-[1-(1-CIA_{\mathrm{PRH}})e^{CIA}{}^{{}_{\mathrm{PRH}}}]\times k_{4}\\ +[1-(1-|Cr|)e^{\left|Cr\right|}]\times k_{5}+k_{0},Cr\in [-1,0] \end{array}\right.$	(7)

**Table 5:** Payoff matrix.

Strategy			Payoff		
Government	Innovation Project Initiator (IPI)	Private Hospitals (PRH)	Government	Innovation Project Initiator (IPI)	Private Hospitals (PRH)
**Supervision**	S1	participation	*-CS*	(1 − $\varphi_{\mathrm{PU}}^{S1}$)*R*_1_ − (1 − $\xi_{\mathrm{PU}}^{S1}$)*C*_1_ − (1 − $\gamma_{\mathrm{PU}}^{S1}$) *RC*_1_	0
	S1	non-participation	*-CS*	(1 − $\varphi_{\mathrm{PU}}^{S1}$)*R*_1_ − (1 − $\xi_{\mathrm{PU}}^{S1}$)*C*_1_ − (1 − $\gamma_{\mathrm{PU}}^{S1}$) *RC*_1_	0
	S2	participation	*GRG-CS*	(1 − $\varphi_{\mathrm{PR}}^{S2}$)*R*_2_ − (1 − $\xi_{\mathrm{PR}}^{S2}$)*C*_2_ − (1 − $\gamma_{\mathrm{PU}}^{S2}$) (*RC*_2_ − *RCRD*)	$\varphi_{\mathrm{PR}}^{S2}$*R*_2_ − $\xi_{\mathrm{PR}}^{S2}$*C*_2_ − $\gamma_{\mathrm{PR}}^{S2}$ (*RC*_2_ − *RCRD*)
	S2	non-participation	*-CS*	(1 − $\varphi_{\mathrm{PU}}^{S1}$)*R*_1_ − (1 − $\xi_{\mathrm{PU}}^{S1}$)*C*_1_ − (1 − $\gamma_{\mathrm{PU}}^{S1}$) *RC*_1_	0
**Non-supervision**	S1	participation	0	(1 − $\varphi_{\mathrm{PU}}^{S1}$)*R*_1_ − (1 − $\xi_{\mathrm{PU}}^{S1}$)*C*_1_ − (1 − $\gamma_{\mathrm{PU}}^{S1}$) *RC*_1_	0
	S1	non-participation	0	(1 − $\varphi_{\mathrm{PU}}^{S1}$)*R*_1_ − (1 − $\xi_{\mathrm{PU}}^{S1}$)*C*_1_ − (1 − $\gamma_{\mathrm{PU}}^{S1}$) *RC*_1_	0
	S2	participation	0	(1 − $\varphi_{\mathrm{PR}}^{S2}$)*R*_2_ − (1 − $\xi_{\mathrm{PR}}^{S2}$)*C*_2_ − (1 − $\gamma_{\mathrm{PU}}^{S2}$)*RC*_2_	$\varphi_{\mathrm{PR}}^{S2}$*R*_2_ − $\xi_{\mathrm{PR}}^{S2}$*C*_2_ − $\gamma_{\mathrm{PR}}^{S2}$*RC*_2_
	S2	non-participation	0	(1 − $\varphi_{\mathrm{PU}}^{S1}$)*R*_1_ − (1 − $\xi_{\mathrm{PU}}^{S1}$)*C*_1_ − (1 − $\gamma_{\mathrm{PU}}^{S1}$) *RC*_1_	0

**Table 6:** Analysis of equilibrium stability.

Equilibrium Point	Non-Negativity of Matrix *J*’s Eigenvalues	Locally Asymptotically Stable	Asymptotically Stable Condition
(0,0,0)	√	Unstable	-
(1,0,0)	-	√	$\begin{array}{c} (1-\varphi_{\mathrm{PU}}^{S1})R_{1}-(1-\xi_{\mathrm{PU}}^{S1})C_{1}-(1-\gamma_{\mathrm{PU}}^{S1})RC_{1}<0;\\ \varphi_{\mathrm{PR}}^{S2}R_{2}-\xi_{\mathrm{PR}}^{S2}C_{2}-\gamma_{\mathrm{PR}}^{S2}RC_{2}<0 \end{array}$
(0,1,0)	√	Unstable	-
(1,1,0)	-	√	$\begin{array}{c} (1-\varphi_{\mathrm{PU}}^{S1})R_{1}-(1-\xi_{\mathrm{PU}}^{S1})C_{1}-(1-\gamma_{\mathrm{PU}}^{S1})RC_{1}-[(1-\varphi_{\mathrm{PR}}^{S2})R_{2}\\ -(1-\xi_{\mathrm{PR}}^{S2})C_{2}-(1-\gamma_{\mathrm{PR}}^{S2})RC_{2}]<0;\\ \varphi_{\mathrm{PR}}^{S2}R_{2}-\xi_{\mathrm{PR}}^{S2}C_{2}-\gamma_{\mathrm{PR}}^{S2}RC_{2}>0;\\ GRG-CS<0 \end{array}$
(0,0,1)	√	Unstable	-
(1,0,1)	√	Unstable	-
(0,1,1)	√	Unstable	-
(1,1,1)	-	√	$\begin{array}{c} (1-\varphi_{\mathrm{PU}}^{S1})R_{1}-(1-\xi_{\mathrm{PU}}^{S1})C_{1}-(1-\gamma_{\mathrm{PU}}^{S1})RC_{1}-[(1-\varphi_{\mathrm{PR}}^{S2})R_{2}\\ -(1-\xi_{\mathrm{PR}}^{S2})C_{2}-(1-\gamma_{\mathrm{PR}}^{S2})RC_{2}+(1-\gamma_{\mathrm{PR}}^{S2})RCRD]<0;\\ \varphi_{\mathrm{PR}}^{S2}R_{2}-\xi_{\mathrm{PR}}^{S2}C_{2}-\gamma_{\mathrm{PR}}^{S2}RC_{2}+(1-\gamma_{\mathrm{PR}}^{S2})RCRD>0;\\ GRG-CS>0 \end{array}$
(*x*’,*y*’,*z*’)	√	Unstable	-

**Table 7:** The values of all parameters in different scenarios.

	Value	Scenario 1	Scenario 2	Scenario 3
Parameter				
*m*0		3	-	-
*m*1		20	-	-
*m*2		10	-	-
*m*3		4.2	-	-
*m*4		2.2	-	-
*m*5		1.9	-	-
*l*0		3.5	-	-
*l*1		4.5	-	-
*l*2		3	-	-
*l*3		2	-	-
*l*4		1	-	1.8
*k*0		4	-	-
*k*1		3	-	16
*k*2		2	-	-
*k*3		1.5	-	0.5
*k*4		0.5	-	-
*k5*		0.6	-	-
*n*		0.5	-	0.1
*u*		0.6	-	-
$\varphi_{\mathrm{PU}}^{S1}$		0.5	-	0.6
$\varphi_{\mathrm{PR}}^{S2}$		0.5	0.3	0.4
$\xi_{\mathrm{PU}}^{S1}$		0.5	-	0.6
$\xi_{\mathrm{PR}}^{S2}$		0.5	0.3	0.4
*CIA*_IPI_,		0.7	-	-
*CIA*_PRH_		0.3	-	-
*CIA*_PUH_		0.8	-	-
*Cr*		0.2	-	-
$\gamma_{\mathrm{PUH}}^{S1}$		0.5	-	-
$\gamma_{\mathrm{PRH}}^{S2}$		0.5	0.6	0.6
GRG		3	-	
CS		1	-	
*RCRD*		1.5	-	-

(The symbol ‘-’ means the value of the parameter is same to this parameter in Scenario 1).
